# Mapping the Germline and Somatic Mutation Interaction Landscape in Indolent and Aggressive Prostate Cancers

**DOI:** 10.1155/2019/4168784

**Published:** 2019-11-13

**Authors:** Tarun Karthik Kumar Mamidi, Jiande Wu, Chindo Hicks

**Affiliations:** ^1^Informatics Institute, University of Alabama at Birmingham, School of Medicine, 1720 2nd Avenue South, Birmingham, AL 35294-3412, USA; ^2^Department of Genetics, Louisiana State University Health Sciences Center, School of Medicine, 533 Bolivar, New Orleans, LA-70112, USA

## Abstract

**Background:**

A majority of prostate cancers (PCas) are indolent and cause no harm even without treatment. However, a significant proportion of patients with PCa have aggressive tumors that progress rapidly to metastatic disease and are often lethal. PCa develops through somatic mutagenesis, but emerging evidence suggests that germline genetic variation can markedly contribute to tumorigenesis. However, the causal association between genetic susceptibility and tumorigenesis has not been well characterized. The objective of this study was to map the germline and somatic mutation interaction landscape in indolent and aggressive tumors and to discover signatures of mutated genes associated with each type and distinguishing the two types of PCa.

**Materials and Methods:**

We integrated germline mutation information from genome-wide association studies (GWAS) with somatic mutation information from The Cancer Genome Atlas (TCGA) using gene expression data from TCGA on indolent and aggressive PCas as the intermediate phenotypes. Germline and somatic mutated genes associated with each type of PCa were functionally characterized using network and pathway analysis.

**Results:**

We discovered gene signatures containing germline and somatic mutations associated with each type and distinguishing the two types of PCa. We discovered multiple gene regulatory networks and signaling pathways enriched with germline and somatic mutations including axon guidance, RAR, WINT, MSP-RON, STAT3, PI3K, TR/RxR, and molecular mechanisms of cancer, NF-kB, prostate cancer, GP6, androgen, and VEGF signaling pathways for indolent PCa and MSP-RON, axon guidance, RAR, adipogenesis, and molecular mechanisms of cancer and NF-kB signaling pathways for aggressive PCa.

**Conclusion:**

The investigation revealed germline and somatic mutated genes associated with indolent and aggressive PCas and distinguishing the two types of PCa. The study revealed multiple gene regulatory networks and signaling pathways dysregulated by germline and somatic alterations. Integrative analysis combining germline and somatic mutations is a powerful approach to mapping germline and somatic mutation interaction landscape.

## 1. Introduction

Prostate cancer (PCa) is the most common noncutaneous cancer in men and one of the leading causes of cancer-related deaths worldwide [1]. It is estimated that 164,690 men were diagnosed with new cases of PCa and 29,430 men died from the disease in 2018 in the United States [1]. The majority of PCas follow the indolent clinical course and do not result in cancer mortality even without treatment. However, a significant proportion of men will develop aggressive tumors that progress rapidly to metastatic disease and require treatment. A key challenge faced by clinicians is distinguishing patients with indolent PCa from patients with aggressive PCa, and identifying patients at high risk of developing aggressive PCa to be prioritized for treatment.

Screening using the prostate-specific antigen (PSA) can detect PCa at earlier, asymptomatic stages, when treatments might be more effective [2, 3]. However, the unintended consequence of increased screening using PSA has been overdiagnosis and overtreatment of PCas which are considered by many experts as indolent and cause no harm [2–4]. Overtreatment of indolent tumors may result in significant morbidity and impaired quality of life for many men. Conversely, many men diagnosed with highly aggressive PCa are undertreated because of the lack of knowledge about which men have the high risk of developing the aggressive form of the disease [2–4]. These concerns led to issuing of a D grade recommendation against PSA-based PCa screening in 2012 by the U.S. Preventive Services Task Force [5, 6]. A review of the evidence by the U.S. Preventive Services Task Force concluded that PSA-based screening results in small or no reduction in prostate cancer-specific mortality and is associated with harms related to subsequent evaluation and treatments, some of which may be unnecessary [5, 6]. Thus, given the controversies, lack of specificity, and inability to accurately identify patients at high risk of developing aggressive PCa using PSA screening, there is an urgent need for (1) a deeper understanding of the genomic differences between indolent and aggressive PCas and (2) discovery of clinically actionable molecular markers dysregulated by genetic alterations, which could be used to improve patient stratification by identifying men at high risk of developing aggressive tumors that could be prioritized for treatment. Such markers could facilitate the realization of precision medicine and could also be used for the development of novel precision prevention strategies.

PCa is a complex disease influenced by both inherited variants in the germline DNA and somatic mutations acquired during formation of the tumors [7, 8]. With the application of high-throughput genotyping over the last two decades, comprehensive catalogues of genetic variants, primarily single-nucleotide polymorphisms (SNPs, herein referred to as germline mutations) and genes associated with an increased risk of developing PCa, have been developed from genome-wide association studies (GWAS) [9–13]. Germline genetic variants discovered from these studies have enabled development of risk prediction models such as polygenic risk scores and polygenic hazard scores to guide screening for PCa [14–16]. At least one polygenic risk score model has been validated for clinical use [17, 18]. However, while polygenic risk scores developed using germline genetic variants have the promise of identifying patients at high risk of developing aggressive cancer, establishing the causal association between genetic susceptibility and tumorigenesis for indolent and aggressive PCas remains a challenge.

With the recent surge of next-generation sequencing and genomic characterization of cancer genomes, discovery of acquired somatic mutations that may drive PCa has come into sharper focus. Large multicenter and multinational projects such as The Cancer Genome Atlas (TCGA) and the International Cancer Genome Consortium (ICGC) have developed comprehensive catalogues of somatic mutations involved in PCa and other cancers [19–21]. Discoveries from these large-scale sequencing studies on cancer genomes have increased our understanding of the molecular taxonomy of PCa [19]. However, while somatic mutations may play a strong role in the development and progression of tumors, emerging evidence indicates that germline genetic variation can contribute to tumorigenesis via diverse mechanisms [7, 8]. Understanding the germline-somatic mutation interaction landscape in indolent and aggressive PCas has the promise of uncovering the molecular causes of aggressive disease, as well as identifying patients at high risk of developing lethal disease to be prioritized for treatment. We recently reported oncogenic interactions and cooperation between genes containing germline and somatic mutations in primary PCa [22] and aggressive PCa [23]. The results from these studies emphasized the relevance of integrating germline with somatic mutation information in PCa biomarker discovery. However, to date, there are no reports on how germline and somatic alterations interact in indolent and aggressive PCas. A deeper understanding of germline-somatic mutation interactions and the genomic differences between indolent and aggressive PCas could potentially improve patient stratification and speed the development of targeted therapies and precision prevention strategies.

The objectives of this study were (1) to discover signatures of germline and somatic mutated genes associated with each type and distinguishing the two types of PCa and (2) to map the germline-somatic mutation interaction landscape in indolent and aggressive PCas and discover the molecular networks and signaling pathways enriched with germline and somatic mutations associated with each type of disease. Our working hypothesis was that genomic alterations in genes containing germline or somatic mutations or a combination thereof could lead to measurable changes distinguishing indolent from aggressive disease. We further hypothesized that PCa originates from a complex interplay between germline and somatic mutations mapped to functionally related oncogenes interacting in gene regulatory networks and signaling pathways which in turn drive indolent and aggressive disease. We addressed these hypotheses using an integrative genomics approach that integrates germline mutation information from GWAS with somatic mutation information from next-generation sequencing on indolent and aggressive PCas from TCGA using gene expression data derived from the same patient samples as the intermediate phenotype. Our modeling approach assumed the gene as the unity of association rather than individual mutations and further assumed that interactions and cooperation between germline and somatic mutations are manifested through gene regulatory networks and signaling pathways.

## 2. Materials and Methods

### 2.1. Source of Germline Mutations and Associated Genes

We used cohort-level information on germline mutations and genes derived from published reports on GWAS. GWAS compared the frequency of common single-nucleotide polymorphisms (SNPs, herein referred to as germline mutations) throughout the entire genomes of PCa patients and controls [24]. GWAS generally evaluated up to one million SNPs in large cohorts of thousands of patients *versus* controls to determine association between SNPs and the probability of developing PCa. Because only 1 or 2 million of approximately 50 million SNPs are assessed, the SNPs associated with PCa through GWAS may not necessarily be the causal genetic risk variants. However, these risk-associated SNPs are segregated from the underlying causal variants, since they are in linkage disequilibrium [25]. Here, we report the efforts of integrating germline with somatic mutation information on indolent and aggressive PCas. Our integrative approach was designed to be all inclusive by using the mutated genes as the units of association rather than individual mutations to address the limitations of GWAS. To address this, we have developed a comprehensive catalogue of germline mutations and genes used in this report and continuously updated it [10, 22, 23, 26, 27]. The details regarding methods of data collection, curation, and annotation, including inclusion and exclusion criteria, have been described in our earlier publications [10, 22, 23, 26, 27] and were based on internationally accepted standards and guidelines proposed by the Human Genome Epidemiology Network for a systematic review of genetic associations [28–32]. The data in our catalogue were supplemented with information from the GWAS catalogue [9–13] which is continuously updated to ensure completeness of the germline variation data used in this study. The resulting data set included 401 genes containing 637 genetic variants associated with an increased risk of developing PCa. It is worth mentioning that the majority of GWAS > 95% were not designed to capture a specific type or subtype of PCa. For this reason, we considered all the genes and genetic variants in each analysis for indolent and aggressive PCas. A complete list of genetic variants and genes along with sources or published reports from which they were derived is presented in Supplementary [Supplementary-material supplementary-material-1] provided as supplementary data to this report.

### 2.2. Somatic Mutation Gene Expression and Clinical Data Sets

We used somatic mutation, gene expression, and clinical data on indolent and aggressive PCas from the TCGA. The data were downloaded from the Genomic Data Commons (GDC; https://gdc-portal.nci.nih.gov/legacy-archive/), a data portal using the data transfer tool [33]. The original data set included 495 samples of prostate adenocarcinoma distributed as 190 indolent samples, 305 aggressive samples, and 52 controls. Because the same TCGA barcode structure is used for both clinical data and molecular data, we used the barcode structure to integrate patient-based clinical data with sample-based somatic mutation and gene expression data. We further processed the data set using gene symbols and somatic mutation information across patient samples. The resulting data set contained somatic mutations and somatic mutated genes in 141 patients with indolent PCa and 188 patients with aggressive PCa. A comprehensive list of somatic mutated genes and the number of events in indolent PCa (sheet-1) and aggressive PCa (sheet-2) obtained from TCGA is presented in Supplementary [Supplementary-material supplementary-material-1]. Gene expression data were derived from the same patient population as somatic mutation and were generated using RNA-seq. After integrating gene expression data with somatic mutation information using clinical information, the resulting data set used in this investigation included 141 samples of patients diagnosed with indolent PCa, 188 samples of patients diagnosed with aggressive PCa, and 52 control samples.

Using clinical information provided by the TCGA consistent with the classification protocols of the American Urological Association [34], we classified the tumors as either indolent or aggressive as described here. In a clinical setting, treatment decisions for PCa patients are guided by various stratification algorithms [34]. Among these parameters, the most potent predictor of PCa mortality is the Gleason grade which ranges from 6 to 10 in the modern era [34]. The presence of Gleason grade ≤6 is associated with very low cancer-specific mortality rates, even in the absence of intervention; therefore, these cancers were classified as indolent in this study. Intermediate-grade disease (Gleason grade 7) has a much more variable clinical course. High Gleason grades 8–10 are aggressive and often lethal tumors and, therefore, were classified as aggressive in this study. Because intermediate-risk tumors with Gleason grade 7 follow a variable clinical course, we considered tumors that scored 3 + 4 favorable intermediate risk and grouped them as low risk (Gleason grade 6). Tumors that scored 4 + 3 were considered as unfavorable intermediate risk and were assigned to tumors with Gleason grades 8–10 (aggressive PCa) consistent with the classification protocols of the American Urological Association [34].

We performed additional data quality control and processing steps on a gene expression data set integrated with mutation information, by imposing filters to remove rows with missing data, such that each row had at least ≥30% data, using the CPM (counts per million) filter (>0.5) implemented in R [35]. The resulting data set was normalized using the trimmed mean of M-values (TMM) normalization method and transformed using voom in the LIMMA package implemented in R [35]. The normalized data contained 18,428 probes and were used in downstream analyses. The probe IDs and gene symbols and names were matched for interpretation using the Ensembl database, a database used for gene annotation in sequencing experiments and sequencing technology platforms.

### 2.3. Data Analysis

**Figure 1:**
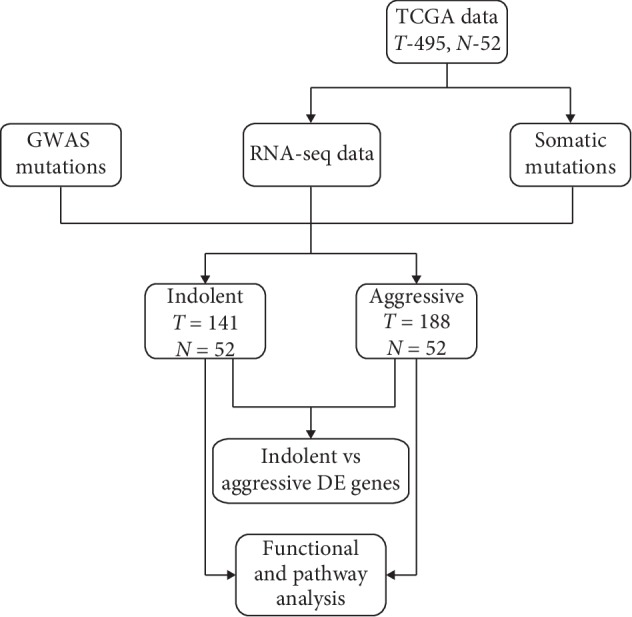
Project design and analysis workflow for integrative analysis combining germline with somatic mutation information on indolent and aggressive cancers using gene expression data as the intermediate phenotype. RNA-seq read count data and somatic information were downloaded from The Cancer Genome Atlas (TCGA). Germline mutation information was manually curated from GWAS and supplemented with information from the GWAS catalogue. The LIMMA (R) package was used for the discovery of differentially expressed (DE) mutated and nonmutated genes. Ingenuity Pathway Analysis (IPA) was used for the discovery of molecular networks and signaling pathways enriched with germline and somatic mutations.

The project design, sources and types of data, and data analysis workflow are presented in Figure 1. After data processing, we compared gene expression levels between patients diagnosed with indolent tumors and matched control samples, and between patients diagnosed with aggressive tumors and matched control samples, using the LIMMA package implemented in R [35] to identify mutated and nonmutated gene signatures associated with each type of PCa.

Subsequently, we compared the expression levels of differentially expressed genes between indolent and aggressive disease to identify mutated and nonmutated genes distinguishing the two patient groups. For each analysis, we used the false discovery rate (FDR) procedure to correct for multiple hypothesis testing by computing the adjusted *p* values [36].

The genes were ranked on adjusted *p* values. Significantly differentially expressed genes were grouped into different categories: genes significantly associated with indolent disease, aggressive disease, or both diseases and genes distinguishing the two types of PCa. The primary focus of this investigation was on germline and somatic mutated genes. Therefore, for the mutated genes significantly associated with indolent and/or aggressive PCa, we performed additional analysis comparing gene expression levels between the two diseases. To ensure that the results are not confounded, for comparing gene expression levels between the two types of PCa, we used only the sets of mutated genes uniquely associated with indolent PCa and those uniquely associated with aggressive PCa. Mutated genes intersecting or significantly associated with both types of PCa were not included in this analysis.

To assess the differences in mutation burden between indolent and aggressive PCas, we quantified the number of somatic mutation events per gene in each type or both types of PCa. We performed this analysis on the sets of genes significantly associated with each type of PCa and genes significantly differentially expressed between indolent and aggressive PCas. A gene was considered highly mutated if it had ≥3 mutation events. From this analysis, we developed comprehensive catalogues of mutated genes and the number of mutation events per gene and used this information to assess the differences in mutation burden between the two types of PCa. To identify genes containing both germline and somatic mutations, we evaluated the 401 genes against all the significantly differentially expressed mutated and nonmutated genes in each type of PCa and between the two types of PCa.

We performed network and pathways analysis separately for indolent and aggressive PCas using the Ingenuity Pathway Analysis (IPA) software to identify molecular networks and biological pathways enriched with germline and somatic mutations [37]. Using IPA, highly significantly differentially expressed genes containing both germline and somatic mutations, germline mutated genes uniquely associated with each disease, and highly somatic mutated genes without germline mutations, but highly significantly associated with each type of PCa, were mapped onto networks and canonical pathways. The networks were trimmed and filtered to include networks with ≥3 connections, to avoid spurious interactions. Our goal was to discover molecular networks and pathways unique to each disease; therefore, genes significantly associated with both types of PCa were not included in this analysis to avoid confounding of the results. For each analysis, the probability score and the log *p* value were calculated to assess the likelihood and reliability of correctly assigning the mutated genes to the correct molecular networks and biological pathways, respectively. A false discovery rate was used to correct for multiple hypothesis testing in pathway analysis. The predicted molecular networks and biological pathways were ranked based on Z-scores and log *p* values, respectively. Gene ontology (GO) [38] analysis as implemented in IPA [37] was performed, to gain insights into the molecular functions, biological processes, and cellular components in which the genes containing germline and somatic mutations are involved and the biological mechanisms through which they are likely to cooperate.

## 3. Results

We integrated germline mutation information from GWAS reports and somatic mutation information from TCGA to map the landscape of oncogenic interactions and cooperation between genes containing germline and somatic mutations, and to discover the molecular networks and signaling pathways dysregulated by these genetic alterations in indolent and aggressive PCas. Here, we report the findings from this innovative approach.

### 3.1. Discovery of Somatic Mutated and Nonmutated Gene Signatures

Our first task was to discover and characterize signatures of somatic mutated and nonmutated genes associated with indolent and aggressive PCas and genes distinguishing the two diseases. To address the issue, we compared gene expression levels between indolent and control samples, between aggressive and control samples, and between the two types of PCa.

Comparison of gene expression levels between indolent and control samples revealed a signature of 10,779 significantly (*p* < 0.05) differentially genes, of which 1,961 (18%) differentially expressed genes had somatic mutations in indolent PCa and 8,818 had no somatic mutations. Comparison of gene expression levels between aggressive and control samples revealed 12,100 significantly (*p* < 0.05) differentially genes, of which 2,498 (21%) differentially expressed genes in aggressive PCa had somatic mutations and 9,602 had no somatic mutations. A complete list of somatic mutated genes significantly associated with indolent and aggressive PCas is presented in Supplementary [Supplementary-material supplementary-material-1]. A complete list of genes without somatic mutations (nonmutated genes) significantly associated with indolent and aggressive PCas is presented in Supplementary [Supplementary-material supplementary-material-1]. There were significant overlaps in significantly differentially expressed mutated and nonmutated genes between the two types of PCa.

**Figure 2:**
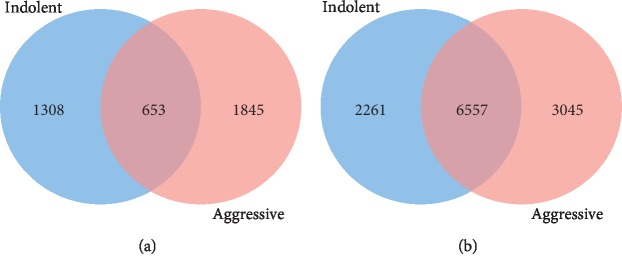
Venn diagrams showing the distribution of somatic mutated genes (a) and nonmutated genes (b) found to be significantly (*p* < 0.05) differentially expressed between case and control samples in indolent and aggressive PCas. Genes in the intersections were found to be differentially expressed in both types of cancer.

To discover gene signatures of somatic mutated and nonmutated genes uniquely associated with each type of PCa, and genes associated with both types of PCa, we evaluated the genes using estimated adjusted *p* values. The distributions of the results between indolent and aggressive tumors for mutated and nonmutated genes are shown in Venn diagrams in Figure 2.

Figure 2(a) shows the distribution of somatic mutated genes in indolent and aggressive tumors. The distribution of nonmutated genes for the two types of PCa is shown in Figure 2(b). The analysis revealed 1,308 somatic mutated genes significantly associated with indolent PCa, 1,845 genes significantly associated with aggressive PCa, and 653 genes significantly associated with both types of PCa (Figure 2(a)). Analysis that focused on genes without somatic mutations revealed 2,261 genes significantly associated with indolent disease, 3,045 genes significantly associated with aggressive disease, and 6,557 genes significantly associated with both types of PCa (Figure 2(b)). These analyses confirmed our hypothesis that genomic alterations in genes containing somatic mutations and genes without somatic mutations could lead to measurable changes associating them with each type and both types of PCa. A complete list of somatic mutated genes significantly associated with indolent PCa only, aggressive PCa only, and both is presented in Supplementary [Supplementary-material supplementary-material-1].

### 3.2. Differences in Gene Expression and Mutation Burden

Overtreatment of indolent tumors may result in significant morbidity and impaired quality of life. Thus, a deeper understanding of the genomic differences between indolent and aggressive PCas was a critical component of this investigation. To address this issue, we evaluated the 1,308 somatic mutated genes significantly associated with indolent PCa and the 1,845 somatic mutated genes significantly associated with aggressive PCa, for the number of mutation events per gene in each type and in both types of PCa. We sought to discover signatures of genes that are mutated and significantly associated with each type of PCa. Genes that were significantly associated with each type of PCa, but mutated in both types of PCa, were grouped separately. This analysis revealed 1,229 genes uniquely mutated and significantly associated with only indolent PCa and 1,697 genes uniquely mutated and significantly associated with only aggressive PCa. In addition, the analysis revealed 79 genes significantly associated with indolent cancer and 148 genes significantly associated with aggressive disease, with somatic mutations in both types of PCa.

To further gain insights into the differences in genomic and somatic alterations between indolent and aggressive PCas, we created a data set combining the 1,229 genes with somatic mutations significantly associated with indolent PCa only and 1,697 genes with somatic mutations significantly associated with aggressive PCa only. We then performed analysis comparing expression levels and the number of somatic mutation events for the genes in the combined data set between indolent and aggressive PCas. Here, we sought to discover signatures of significantly differentially expressed genes which are also differentially mutated between indolent and aggressive PCas. Therefore, genes with somatic mutations in both types of PCa were not included in this analysis.

The analysis revealed a signature of 970 significantly (*p* < 0.05) differentially expressed genes distinguishing indolent from aggressive tumors. This included the 394 genes with somatic mutations in indolent PCa only and the 576 genes with somatic mutations in aggressive PCa only. This confirmed our hypothesis that, for a selected set of genes, there are differences in mutation burden and gene expression between indolent and aggressive PCas. The results showing the most highly mutated (>3 mutation events per gene) genes significantly differentially expressed and differentially mutated between indolent and aggressive PCas are presented in Table 1.

There was significant variation in the number of somatic mutations per gene in each type of PCa. In both types of PCa, the number of somatic mutation events per gene varied from 1 to 5. The genes *FOXP1*, *PAPPA*, *FLRT2*, *LMO7*, *DPYSL3*, *RAI14*, *SIK3*, *DAAM2*, *MYOM1*, *SLIT1*, *MOAP1*, *MAML3*, *NES*, *CBX4*, *and METTL3* had mutations in indolent PCa only (Table 1). The genes *EPHB1*, *KIAA1614*, *SACS*, *SMAD4*, *PCDHA1*, *TNS1*, *CACNA1C*, *DEPDC1*, *PCDHGA9*, *LRP4*, *KLHL2*, *CDC20*, *ARHGEF39*, *CGNL1*, *SKIV2L2*, *FAM196A*, *IL6ST*, *ATP2B4*, *TGFBR3*, *TIGD3*, *NOS1*, *SRSF2*, *MYO9A*, *KIF13A*, *UBR3*, *WIF1*, *LRGUK*, *ERBB4*, and *NYNRIN* had mutations in aggressive PCa only (Table 1). A complete list of genes that are somatic mutated in each type of PCa and significantly differentially expressed between the two types of PCa is presented in Supplementary [Supplementary-material supplementary-material-1].

### 3.3. Discovery of Gene Signatures Enriched with Germline and Somatic Mutations

**Figure 3:**
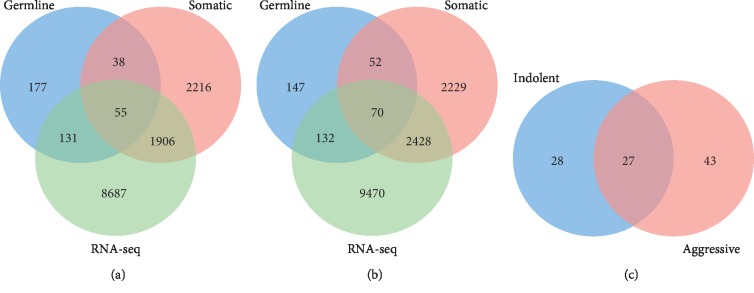
Venn diagram showing the distribution of genes containing both germline and somatic mutations, germline mutations only, and somatic mutations only in (a) indolent PCa and (b) aggressive PCa. (c) Venn diagram showing the overlap of genes containing both germline and somatic mutations in both disease types.

To begin to link genetic susceptibility with tumorigenesis and to infer the potential causal association between the gene expression and each type of PCa, we performed several analysis strategies. First, we evaluated the 401 genes containing germline mutations for somatic mutations, to address the hypothesis that genes containing germline mutations also harbor somatic mutations. Second, we evaluated the 401 genes containing germline mutations for association with each type of PCa using *p* values computed from gene expression data. Third, we evaluated the germline mutated genes for differences in expression levels and the number of mutation events per gene between indolent and aggressive PCas. The distributions of the results from these analyses are presented in Venn diagrams in Figure 3 for each type and both types of PCa.

The results for indolent PCa are presented in Figure 3(a). Out of the 401 genes containing germline mutations evaluated, 93 genes contained both germline and somatic mutations. From this number, 55 genes were significantly associated with indolent PCa (Figure 3(a)). In addition, the analysis revealed a signature of 131 genes containing only germline mutations significantly associated with indolent PCa. The remaining 177 genes contained only germline mutations and were not significantly associated with the disease. A complete list of germline mutated genes significantly associated with indolent PCa is presented in Supplementary [Supplementary-material supplementary-material-1].

The results for aggressive PCa are presented in Figure 3(b). Out of the 401 genes containing germline mutations evaluated, 122 genes contained both germline and somatic mutations, of which 70 genes were significantly associated with aggressive PCa. In addition, the analysis revealed a signature of 132 genes containing only germline mutations significantly associated with aggressive PCa. The remaining 147 genes contained only germline mutations and were not significantly associated with the disease. In both indolent and aggressive PCas, there was significant variation in the distribution of somatic mutations among the genes containing germline mutations. A complete list of germline mutated genes significantly associated with aggressive PCa is presented in Supplementary [Supplementary-material supplementary-material-1].

To address the hypothesis that genes containing both germline and somatic mutations significantly associated with each type of PCa are unique to each type of PCa, we evaluated the 55 genes significantly associated with indolent PCa and the 70 genes significantly associated with aggressive PCa using the estimates of *p* values computed as described in Materials and Methods. Here, we sought to discover genes significantly associated with each type of PCa and genes associated with both types of PCa.

The results of this evaluation are presented in Figure 3(c). The analysis revealed a signature of 28 genes significantly associated with indolent PCa, of which 23 genes were only mutated in indolent PCa and 5 in both types of PCa. The results showing a signature of the 23 genes containing both germline and somatic mutations significantly associated with and only somatic mutated in indolent PCa are presented in Table 2. There were significant variation and sparseness in the number of both germline and somatic mutations per gene. The most germline mutated gene was *TNRC6B*, whereas the most somatic mutated genes were *TMPRSS2* and *MAML3*.

Evaluation that focused on aggressive PCa (Figure 3(c)) revealed 43 genes significantly associated with aggressive PCa, of which 38 genes were only somatic mutated in aggressive PCa, whereas 5 genes were somatic mutated in both. In addition, the analysis revealed 27 genes containing germline and somatic mutations significantly associated with both types of PCa (Figure 3(c)). The results showing the 38-gene signature enriched with both germline and somatic mutations and somatic mutated only in aggressive PCa are presented in Table 3. There were significant variation and sparseness in the number of both germline and somatic mutations per gene. The most germline mutated genes were *SLC22A3*, *KLK2*, *RNASEL*, *POU5F1B*, and *TBX5*, whereas the most somatic mutated genes were *KIF13A* and *ZNF827*.

### 3.4. Differences in Somatic Mutations between Indolent and Aggressive PCas for Genes Containing Both Germline and Somatic Mutations

One of the objectives of this study was to investigate whether there are differences in expression levels and the number of somatic mutation events among the genes containing both germline and somatic mutations between indolent and aggressive PCas. To address this hypothesis, we created a new data set combining the 23 genes containing both germline and somatic mutations significantly associated with and somatic mutated in indolent PCa only with the 38 genes containing both germline and somatic mutations significantly associated with and somatic mutated in aggressive PCa only (Figure 3(c)). We then compared the expression levels of the 61 genes in the combined data set between indolent and aggressive PCas. The analysis produced a signature of 29 significantly (*p* < 0.05) differentially expressed and differentially somatic mutated genes distinguishing indolent from aggressive disease. The results showing the 29-gene signature are presented in Table 4. Out of the 29 significantly differentially expressed and differentially somatic mutated genes containing both germline and somatic mutations and distinguishing indolent from aggressive disease, 12 genes had somatic mutations in indolent PCa only. The other 17 genes had somatic mutations in aggressive PCa only. The frequency of somatic mutations was higher in aggressive tumors than in indolent tumors.

Overall, the analysis of germline and somatic mutation patterns in indolent and aggressive PCas produced evidence that genes containing germline mutations also harbor somatic mutations. Some genes had mutations in indolent PCa only. Others had mutations in aggressive PCa only. We also found evidence of genes mutated in both types of PCa. The somatic mutations in genes containing germline mutations were remarkably heterogeneous, and the somatic mutation profiles were sparse. This could partially be explained by the heterogeneity of the disease.

### 3.5. Mapping the Germline-Somatic Mutation Interactions Using Network and Pathway Analysis

One of the primary objectives of this study was to map the germline and somatic mutation interaction landscape in indolent and aggressive PCas, and to discover signaling pathways enriched with germline and somatic mutations. Such information would provide insights into the biological mechanisms through which the germline and somatic genomes cooperate to drive the disease and shape the phenotypes. Our working hypothesis was that indolent and aggressive PCas originate from a complex interplay between genes containing germline and somatic mutations and that these complex arrays of interacting genetic factors affect entire molecular networks and signaling pathways which in turn drive the disease and shape the observed clinical phenotypes as either indolent or aggressive.

To address this hypothesis, we performed network and pathway analysis separately for each type of PCa as explained in Materials and Methods. For indolent PCa, we used the 28 genes containing both germline and somatic mutations that were significantly associated with indolent PCa, the 43 genes containing germline mutations only that were highly significantly associated with indolent PCa, and the 216 genes with high somatic mutation events that were significantly associated with indolent PCa. Likewise, for aggressive PCa, we used the 43 genes containing both germline and somatic mutations that were significantly associated with aggressive PCa, the 44 genes containing germline mutations only that were significantly associated with aggressive PCa, and the 343 genes with high somatic mutation events that were significantly associated with aggressive PCa.

The scientific premise and rationale for using highly somatic mutated genes with germline mutations only associated with each disease, in addition to genes containing both germline and somatic mutations in network and pathway analysis, were to overcome some of the limitations inherent in GWAS as mentioned earlier in Materials and Methods and elucidated here. GWAS discoveries explain only a small proportion of the phenotypic variation. Crucially, most of the genetic variants from GWAS reported thus far have undefined functions, are not PCa-type specific, and have not been causally associated with PCa. Thus, limiting the analysis to only genes containing both germline and somatic mutations associated with each type of PCa could miss important gene regulatory networks and signaling pathways with somatic mutations driving the two diseases.

**Figure 4:**
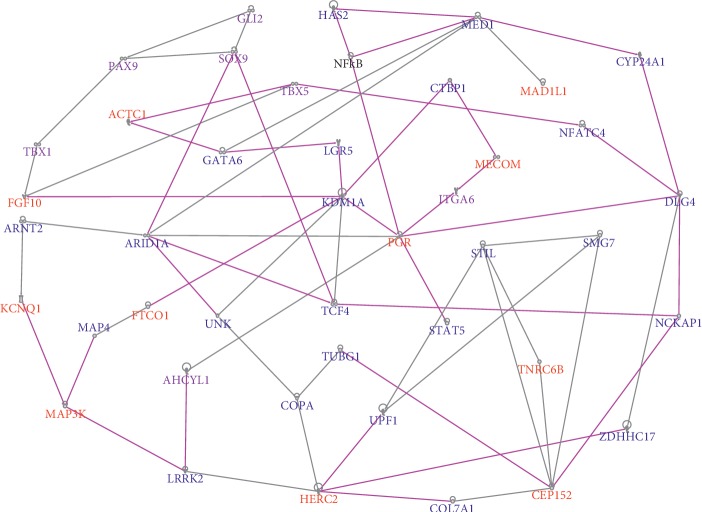
Molecular networks showing oncogenic interactions and cooperation between genes containing germline mutations in indolent PCa. Genes in red font contain both germline and somatic mutations, genes in purple font contain only germline mutations, and genes in blue font have the highest somatic mutation events. All genes were significantly associated with indolent PCa only as determined by gene expression data and somatic mutations. The line colors indicate overlap in molecular functions with multiple networks.

The results of network analysis for indolent PCa are presented in Figure 4. Network analysis revealed molecular networks enriched with germline and somatic mutations confirming our hypothesis that genes containing germline and somatic mutations are functionally related and interact in complex gene regulatory networks. Network analysis revealed 20 gene regulatory networks enriched with both germline and somatic mutations with *Z*-scores ranging from 2 to 51. The top 6 networks (i.e., *Z*-score ≥ 29) were merged using IPA's network merge module and are presented in Figure 4. The top networks contained genes predicted to be involved in organismal survival, cellular movement, and cell death and survival (*Z*-score 51), organismal development (*Z*-score 43), cellular movement and morphology (*Z*-score 43), DNA replication, recombination, and repair and cancer (*Z*-score 38), cancer, connective tissue disorders, and organismal injury and abnormalities (*Z*-score 36), and cancer, connective tissue disorders, and developmental disorder (*Z*-score 29) (see Supplementary [Supplementary-material supplementary-material-1] for additional networks with lesser *Z*-scores).

The remainder of the networks contained genes predicted to be involved in overlapping molecular functions, cellular assembly and organization, cellular function and maintenance, tissue development, cancer, amino acid metabolism, molecular transport, cell cycle, cellular assembly and organization, cellular function and maintenance, cell death and survival, posttranslational modification, and renal and urological disease. A complete list of all the 20 predicted networks and germline-somatic genes mapping to those networks including molecular functions in which the genes are involved is presented in Supplementary [Supplementary-material supplementary-material-1].

Pathway analysis revealed signaling pathways enriched with germline and somatic mutations, many of which have been implicated in PCa including axon guidance; adipogenesis; RAR, GP6, thrombin, WINT, MSP-RON, STAT3, PI3K, and TR/RxR activation; and molecular mechanisms of cancer, NF-KB, prostate cancer, GP6, androgen, and VEGF signaling pathways. The top upstream regulators included *CTNNB1*, *ITGB1*, and *SMO*.

**Figure 5:**
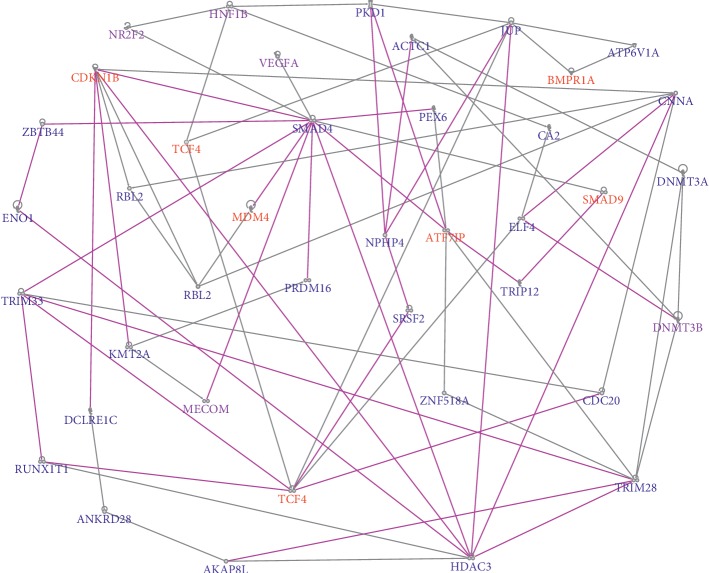
Molecular networks showing oncogenic interactions and cooperation between genes containing germline mutations in aggressive PCa. Genes in red font contain both germline and somatic mutations, genes in purple font contain only germline mutations, and genes in blue font have the highest somatic mutation events. The line colors indicate overlap in molecular functions with multiple networks.

The results of network analysis for aggressive PCa are presented in Figure 5. Network analysis revealed molecular networks enriched with germline and somatic mutations confirming our hypothesis that oncogenic interactions and cooperation between and among genes containing germline and somatic mutations are likely to occur in gene regulatory networks. Network analysis revealed 25 gene regulatory networks enriched with both germline and somatic mutations with *Z*-scores ranging from 8 to 49. The top 6 networks (i.e., *Z*-score ≥ 28) were merged and are presented in Figure 5. The top networks included genes predicted to be involved in DNA replication, recombination, and repair and gene expression (*Z*-score 49), hereditary disorder (*Z*-score 39), cell cycle, embryonic development, and cancer (*Z*-score 35), organismal development and skeletal and muscular system development and function (*Z*-score 35), cancer (*Z*-score 30), and RNA damage and repair, RNA posttranscriptional modification, and cellular development (*Z*-score 28).

The remainder of the networks contained genes predicted to be involved in overlapping molecular functions, including embryonic development, cancer, organismal injury and abnormalities, cell-to-cell signaling and interaction, organismal development, organismal functions, cell death and survival, connective tissue development and function, carbohydrate metabolism, immunological disease, cell morphology, cell cycle, cellular assembly and organization, cellular function and maintenance, inflammatory disease, cancer, cell cycle and cellular development, and reproductive system development and function. A complete list of all the 25 predicted networks and germline-somatic genes mapping to those networks including molecular functions in which the genes are involved is presented in Supplementary [Supplementary-material supplementary-material-1].

Pathway analysis revealed signaling pathways enriched with germline and somatic mutations, many of which have been implicated in PCa including axon guidance; adipogenesis; MSP-RON, RAR, and GP6 activation; and molecular mechanisms of cancer, NF-KB, prostate cancer, GP6, androgen, and VEGF signaling pathways. The top upstream regulators included *TGFB1* and *GLI1.* There were some overlaps in pathways between indolent and aggressive disease. There were some overlaps in signaling pathways involved in the two types of PCa.

Interestingly, for both indolent and aggressive PCas, network and pathway analysis including highly somatic mutated genes revealed interactions and functional relationships between the highly somatic mutated genes and the genes containing germline mutations, confirming our hypothesis that focusing only on genes containing both germline and somatic mutations could miss important gene regulatory networks and signaling pathways. Overall, the investigation revealed that oncogenic interactions and cooperation between genes containing germline and somatic mutations occur through complex gene regulatory networks and signaling pathways.

## 4. Discussion

We report a novel and innovative integrative genomic approach to mapping the landscape of oncogenic interactions and cooperation between germline and somatic mutated genes in indolent and aggressive PCas. Our investigation was driven by the expectation that (1) mapping oncogenic interactions between germline and somatic mutations in indolent and aggressive PCas could lead to understanding of how the germline and somatic genomes cooperate during tumorigenesis and (2) a deeper understanding of genomic differences between indolent and aggressive PCas could improve patient stratification and identification of patients at high risk of developing aggressive PCa to be prioritized for treatment. There are several innovative aspects and clinical relevance of our approach, and the results from this investigation are summarized as follows:

1. Mapping the landscape of oncogenic interactions in indolent and aggressive PCas: to date, analysis distinguishing indolent from aggressive PCa has focused on using transcriptome data to understand the molecular taxonomy and to discover molecular signatures distinguishing indolent from aggressive disease [8]. Here, we found evidence that genes containing germline mutations also harbor somatic mutations and interact in gene regulatory networks and signaling pathways. This suggests that germline-somatic mutations may cooperate and drive PCa phenotypes in two ways: (1) through gene regulatory networks and (2) through signaling pathways. Although we did not investigate the impact of germline mutations on the somatic genome, it has been reported that alterations in the germline genome potentiate the development of acquired somatic driver mutations by mediating the effects of specific functionally related oncogenes [7, 8].
2. Bridging precision medicine with precision prevention: the identification of molecular drivers of PCa such as somatic mutations used in this study is critical for precision oncology [39]. Likewise, germline mutations used in this study could be used to identify PCa patients at high risk of developing aggressive PCa, a critical step in the realization of precision prevention [40]. The novel and innovative aspect of this investigation is that it bridges precision medicine with precision prevention by linking genetic susceptibility with tumorigenesis. Indeed, multiple advanced algorithms to identify somatic driver mutations and predict outcomes now exist [39, 41]. Our study adds a new dimension by integrating germline with somatic mutation information on indolent and aggressive PCas using gene expression data as the intermediate phenotype. To our knowledge, this is the first study to report such findings.
3. Risk prediction: prevention is the holy grail of cancer elimination [40]. The results of this investigation provide foundational knowledge on how information on germline mutations associated with an increased risk of developing PCa currently used in developing polygenic risk scores [14–18] can be optimally leveraged and integrated with somatic mutation information to link genetic susceptibility to tumorigenesis. The novel aspect of this finding is that while polygenic risk scores have relied on genetic variants alone, leveraging polygenic scores with somatic mutation information could lead to development of more innovative dual-purpose models for predicting both risk and outcomes.
4. Distinguishing indolent from aggressive tumors in PCa: a critical unmet medical need by clinicians is lack of molecular markers with specificity and sensitivity to accurately distinguish indolent from aggressive tumors. Discoveries from this investigation have demonstrated that integrative analysis provides a framework for a deeper understanding of the genomic differences between indolent and aggressive disease. The discovery of differentially mutated genes which are also differentially expressed distinguishing indolent from aggressive PCa in this study suggests that patient risk stratification may be amenable to mutation-based classification, or a combination of mutation and transcriptome data. This finding has not been previously reported.
5. Discovery of potential therapeutic targets: an important and innovative aspect of this investigation is the discovery of molecular networks and signaling pathways enriched with germline and somatic mutations. These discoveries provide insights into the broader biological context in which interactions and cooperation between germline and somatic mutations are likely to occur. Crucially, they elucidate the potential drug targets dysregulated by germline and somatic alterations, which could be used in the development of novel therapeutics.

### 4.1. Limitations of the Study

Although the study shows great promise in mapping oncogenic interactions and cooperation between germline and somatic mutations, there are several limitations that we outline and acknowledge herein. We are aware of the limitations of GWAS discoveries and sequencing and transcriptome studies. The majority of the germline mutations from GWAS studies used in this investigation map to intronic regions of the genes, and their functions have not been defined. The integration used here relied on GWAS discoveries from diverse clinical phenotypes; thus, they lack specificity to indolent and aggressive disease. Validating the germline mutations in indolent and aggressive PCas would provide additional insights not captured in this investigation.

A although we did not investigate the functions of germline and somatic mutations in this study, we have previously shown that germline mutations from GWAS disrupt regulatory sites and regions such as enhancer elements and binding and splice sites [42], suggesting that they may have a functional role. Importantly, network and pathway analysis used in this study addresses some of the limitations. Another limitation is that both germline and somatic mutation information and gene expression data used in this study were derived mainly from men of European ancestry. Although some of the genetic variants discovered using GWAS thus far can be generalized to multiple populations [43], genetic variants can vary among populations and can confer population-specific risks to PCa [43, 44]. Moreover, gene expression can differ between populations [45, 46]. Thus, using diverse ethnic populations is needed if the genomic revolution and its offshoots of precision medicine and precision prevention are to benefit the population equitably and not exacerbate health disparities in PCa. Nevertheless, despite these limitations, discoveries from this investigation provide useful information about the possible oncogenic interactions and cooperation between genes containing germline and somatic mutations in indolent and aggressive PCas. If validated, the new biomarkers discovered in this study have the potential to facilitate the realization of precision medicine and precision prevention in PCa. Given the limitation of GWAS discoveries outlined here, our future research work will focus on validating germline mutations in indolent and aggressive PCas in different populations, and leveraging information on genetic variants with somatic mutation and gene expression to develop more robust risk and outcome prediction models. It is worth noting that although our focus here was PCa, our approach is applicable to other cancers and common human diseases.

## 5. Conclusions

We report interactions and cooperation between genes containing germline and somatic mutations in indolent and aggressive PCas. The investigation shows that interactions and cooperation between germline and somatic mutations are likely to occur through gene regulatory networks and signaling pathways. The results also revealed differences in somatic mutation events and gene expression between indolent and aggressive PCas. The results highlight the need for integrating germline and somatic mutations for the discovery of molecular markers and potential drug targets in PCa.

## Figures and Tables

**Table 1:** List of the topmost highly somatic mutated genes in indolent PCa only and aggressive PCa only that were significantly differentially expressed between the two types of PCa.

Genes	Cytoband	Adj. *p* value	Number of somatic events	
			Indolent	Aggressive
*FOXP1*	3p13	0.012103	5	
*PAPPA*	9q33.1	0.031604	4	
*FLRT2*	14q31.3	6.56*E* − 06	3	
*LMO7*	13q22.2	0.000281	3	
*DPYSL3*	5q32	0.001084	3	
*RAI14*	5p13.2	0.002896	3	
*SIK3*	11q23.3	0.003014	3	
*DAAM2*	6p21.2	0.003032	3	
*MYOM1*	18p11.31	0.009598	3	
*SLIT1*	10q24.1	0.014083	3	
*MOAP1*	14q32.12	0.014239	3	
*MAML3*	4q31.1	0.01755	3	
*NES*	1q23.1	0.021829	3	
*CBX4*	17q25.3	0.032005	3	
*METTL3*	14q11.2	0.0396	3	
*EPHB1*	3q22.2	0.003026		5
*KIAA1614*	1q25.3	0.024418		5
*SACS*	13q12.12	0.0256		5
*SMAD4*	18q21.2	0.000668		4
*PCDHA1*	5q31.3	0.000715		4
*TNS1*	2q35	0.001366		4
*CACNA1C*	12p13.33	0.001809		4
*DEPDC1*	1p31.3	0.002686		4
*PCDHGA9*	5q31	0.005125		4
*LRP4*	11p11.2	0.006512		4
*KLHL2*	4q32.3	0.044345		4
*CDC20*	1p34.2	2.20*E* − 10		3
*ARHGEF39*	9p13.3	4.82*E* − 10		3
*CGNL1*	15q21.3	7.31*E* − 07		3
*SKIV2L2*	q11.2	2.43*E* − 06		3
*FAM196A*	10q26.2	2.94*E* − 05		3
*IL6ST*	5q11.2	5.74*E* − 05		3
*ATP2B4*	1q32.1	0.000125		3
*TGFBR3*	1p22.1	0.000281		3
*TIGD3*	11q13.1	0.000404		3
*NOS1*	12q24.22	0.000986		3
*SRSF2*	17q25.2	0.002276		3
*MYO9A*	15q23	0.003854		3
*KIF13A*	6p22.3	0.004177		3
*UBR3*	2q31.1	0.004346		3
*WIF1*	12q14.3	0.015825		3
*LRGUK*	7q33	0.03038		3
*ERBB4*	2q34	0.045989		3
*NYNRIN*	14q12	0.047476		3

**Table 2:** List of 23 genes containing both germline and somatic mutations significantly associated with indolent PCa only.

Genes	GWAS				RNA-seq	
	Region	Most significant SNP_ID	*p* value	Germline mutation events	Expression *p* value	Somatic events (indolent)
*TMPRSS2*	21q22.3	rs1041449	3.00*E* − 08	1	3.89*E* − 05	3
*MAML3*	4q28	rs736349	0.002	1	3.72*E* − 11	3
*MECOM*	3q26.2	rs142436749	5.00*E* − 09	1	6.41*E* − 17	2
*TNRC6B*	22q13.1	rs58133635	5.00*E* − 12	7	0.000913	1
*MN1*	22q12.1	rs6005451	4.00*E* − 06	2	0.000669	1
*FYCO1*	3p21.3	rs1545985	6.61*E* − 06	2	0.000649	1
*CLVS2*	6q22.31	rs13192613	3.00*E* − 06	2	1.66*E* − 06	1
*IRX4*	5p15.33	rs12653946	3.90*E* − 18	2	1.09*E* − 09	1
*ACTC1*	8q24.21	rs6983267	4.00*E* − 06	2	3.78*E* − 11	1
*WWOX*	16q23.3	rs11150069	9.43*E* − 06	1	0.018357	1
*MAD1L1*	7p22.3	rs527510716	5.00*E* − 08	1	0.017161	1
*NR2F2*	15q26.2	rs11637980	2.00*E* − 06	1	0.007414	1
*IL1RAPL1*	Xp22.1	rs225061	9.71*E* − 04	1	0.00361	1
*BCAS1*	20q13.2	rs6091758	6.00*E* − 18	1	0.000156	1
*KCNQ1*	11p15.5	rs231362	0.01	1	0.000153	1
*GALNTL6*	4q34.1	rs494770	0.01	1	0.000109	1
*FGF10*	5p12	rs2121875	1.00*E* − 08	1	1.87*E* − 06	1
*ZBTB38*	3q23	rs1991431	3.00*E* − 11	1	9.04*E* − 07	1
*LARP4B*	10p15.3	rs141536087	9.00*E* − 13	1	1.15*E* − 07	1
*COL23A1*	5q35.3	rs4976790	7.00*E* − 09	1	5.14*E* − 08	1
*FAM111B*	11q12.1	rs1938781	1.10*E* − 10	1	4.53*E* − 09	1
*MLPH*	2q37.3	rs2292884	4.00*E* − 11	1	1.38*E* − 13	1
*FAM111A*	11q12.1	rs1938781	1.10*E* − 10	1	4.89*E* − 16	1

**Table 3:** List of 38 genes containing both germline and somatic mutations specific to aggressive PCa.

Genes	GWAS				RNA-seq	
	Region	Most significant SNP_ID	*p* value	Germline mutation events	Expression *p* value	Somatic events (aggressive)
*KIF13A*	6p22.3	rs10456809	5.00*E* − 06	2	0.041976	3
*ZNF827*	4q31.22	rs56935123	4.00*E* − 09	2	0.004524	3
*TRIM31*	6p22.1	rs7767188	2.00*E* − 08	2	1.43*E* − 08	2
*FARP2*	2q37.3	rs3771570	1.00*E* − 14	1	0.013316	2
*KIAA1211*	4q12	rs629242	7.25*E* − 07	1	0.006964	2
*TBX3*	12q24.21	rs11067228	1.00*E* − 14	1	0.001996	2
*MDM4*	1q32.1	rs4245739	3.00*E* − 24	1	0.000471	2
*GLI2*	2q14	rs11122834	5.00*E* − 06	1	9.54*E* − 07	2
*UHRF1BP1*	6p21.31	rs9469899	5.00*E* − 09	1	5.03*E* − 09	2
*SIX1*	14q23.1	rs7153648	2.00*E* − 09	1	2.53*E* − 17	2
*SLC22A3*	6q25.3	rs4646284	3.20*E* − 52	4	2.88*E* − 07	1
*KLK2*	19q13.33	rs2735839	6.00*E* − 37	4	1.49*E* − 14	1
*RNASEL*	1q25	rs486907	0.004	3	5.01*E* − 17	1
*POU5F1B*	8q24.21	rs1447295	4.00*E* − 23	3	4.16*E* − 18	1
*TBX5*	12q24.21	rs1270884	1.00*E* − 18	2	1.49*E* − 05	1
*ADGRG1*	16q21	rs11863709	2.00*E* − 11	2	1.03*E* − 05	1
*SLC19A2*	1q23.3	rs3765227	1.26*E* − 04	2	3.62*E* − 12	1
*PDLIM5*	4q22.3	rs17021918	1.00*E* − 24	2	2.99*E* − 20	1
*EPHA10*	1p34.3	rs731174	5.00*E* − 06	2	3.32*E* − 44	1
*PHF20L1*	8q24.22	rs2472537	2.12*E* − 04	1	0.036862	1
*CDYL*	6p25.1	rs79774606	9.00*E* − 06	1	0.011624	1
*MYO9B*	19p13.11	rs11666569	8.00*E* − 09	1	0.005102	1
*SHROOM2*	Xp22.2	rs2405942	2.00*E* − 12	1	0.00286	1
*PKNOX2*	11q24.2	rs138466039	2.00*E* − 11	1	0.002475	1
*ADNP*	20q13.13	rs12480328	5.00*E* − 11	1	0.000235	1
*KCNN3*	1q21.3	rs1218582	1.00*E* − 08	1	1.06*E* − 05	1
*ITGA6*	2q31.1	rs12621278	2.00*E* − 42	1	4.86*E* − 06	1
*SMAD9*	13q13.3	rs140971918	4.00*E* − 06	1	1.45*E* − 06	1
*TCF4*	18q21.2	rs28607662	3.00*E* − 08	1	1.35*E* − 07	1
*TBX1*	22q11.21	rs2238776	2.00*E* − 08	1	4.61*E* − 08	1
*KLHL15*	Xp22.11	rs6627995	1.00*E* − 13	1	1.60*E* − 08	1
*BMPR1A*	10q22.3	rs11597689	0.03	1	2.78*E* − 09	1
*TTC7A*	2p16.3	rs10194115	5.00*E* − 07	1	3.66*E* − 10	1
*EBF2*	8p21.2	rs11135910	9.00*E* − 13	1	1.83*E* − 10	1
*FTO*	16q12.2	rs9939609	0.04	1	6.25*E* − 11	1
*B3GAT1*	11q25	rs878987	5.00*E* − 08	1	4.02*E* − 12	1
*EIF2S3*	Xp22.11	rs6627995	1.00*E* − 13	1	1.49*E* − 13	1
*FERMT2*	14q22.1	rs8008270	6.00*E* − 16	1	2.14*E* − 24	1

**Table 4:** List of 29 genes containing both germline and somatic mutations and DE in indolent PCa (Ind) and aggressive PCa (Agg).

Genes	GWAS				Somatic events		RNA-seq
	Region	Most significant SNP_ID	*p* value	Mutation events	Ind	Agg	Adj. *p* value
*MAML3*	4q28	rs736349	0.002	1	3		0.01755
*TNRC6B*	22q13.1	rs58133635	5.00*E* − 12	7	1		0.008588
*ACTC1*	15q14	rs6983267	4.00*E* − 06	2	1		4.81*E* − 05
*CLVS2*	6q22.31	rs13192613	3.00*E* − 06	2	1		0.009268
*FYCO1*	3p21.3	rs1545985	6.61*E* − 06	2	1		0.014872
*COL23A1*	5q35.3	rs4976790	7.00*E* − 09	1	1		7.09*E* − 07
*FGF10*	5p12	rs2121875	1.00*E* − 08	1	1		8.43*E* − 05
*FAM111B*	11q12.1	rs1938781	1.10*E* − 10	1	1		0.000116
*BCAS1*	20q13.2	rs6091758	6.00*E* − 18	1	1		0.000187
*LARP4B*	10p15.3	rs141536087	9.00*E* − 13	1	1		0.008236
*ZBTB38*	3q23	rs1991431	3.00*E* − 11	1	1		0.022455
*WWOX*	16q23.3	rs11150069	9.43*E* − 06	1	1		0.039678
*KIF13A*	6p22.3	rs10456809	5.00*E* − 06	2		3	0.004177
*TRIM31*	6p22.1	rs7767188	2.00*E* − 08	2		2	0.025711
*TBX3*	12q24.21	rs11067228	1.00*E* − 14	1		2	0.0225
*MDM4*	12q15	rs4245739	3.00*E* − 24	1		2	0.027709
*SLC22A3*	6q25.3	rs4646284	3.20*E* − 52	4		1	3.08*E* − 08
*SLC19A2*	1q23.3	rs3765227	1.26*E* − 04	2		1	0.00309
*ADGRG1*	16q21	rs11863709	2.00*E* − 11	2		1	0.029341
*TBX5*	12q24.21	rs1270884	1.00*E* − 18	2		1	0.032995
*B3GAT1*	11q25	rs878987	5.00*E* − 08	1		1	5.30*E* − 05
*PKNOX2*	11q24.2	rs138466039	2.00*E* − 11	1		1	0.000584
*SMAD9*	13q13.3	rs140971918	4.00*E* − 06	1		1	0.001413
*FERMT2*	14q22.1	rs8008270	6.00*E* − 16	1		1	0.003022
*KCNN3*	1q21.3	rs1218582	1.00*E* − 08	1		1	0.007503
*ITGA6*	2q31.1	rs12621278	2.00*E* − 42	1		1	0.008034
*FTO*	16q12.2	rs9939609	0.04	1		1	0.008509
*KLHL15*	Xp22.11	rs6627995	1.00*E* − 13	1		1	0.035398
*CDYL*	6p25.1	rs79774606	9.00*E* − 06	1		1	0.041002

## Data Availability

This study used publicly available deidentified data. GWAS data are provided in Supplementary [Supplementary-material supplementary-material-1]. Additional GWAS information is available in the GWAS catalogue managed by the European Bioinformatics Institute (https://www.ebi.ac.uk/gwas/). Original gene expression and mutation data are available in the TCGA via the Genomics Data Commons (https://gdc.cancer.gov/). Additional data on mutated and nonmutated genes associated with and distinguishing the two diseases are provided in supplementary tables in this paper.

## Supplementary Materials

Supplementary Materials Supplementary Table SA: a comprehensive list of single-nucleotide polymorphisms (herein called genetic variants) and genes associated with increased risk of developing prostate cancer and published GWAS reports denoted by the PubMed ID and actual reference from which the data were extracted. Supplementary Table S1A: a complete list of somatic mutated genes found to be significantly differentially expressed in indolent (sheet-1) and aggressive (sheet-2) tumors matched with controls. Supplementary Table S1B: a complete list of nonsomatic mutated genes found to be significantly differentially expressed in indolent (sheet-1) and aggressive (sheet-2) tumors matched with controls. Supplementary Table S2: a comprehensive list of somatic mutated genes in indolent (sheet-1) and aggressive (sheet-2) PCa obtained from TCGA. Supplementary Table S2A: a complete list of somatic mutated genes found to be significantly differentially expressed in only indolent PCa, only aggressive PCa, and both tumors. Supplementary Table S1: a complete list of significantly differentially expressed somatic mutated genes distinguishing patients with indolent PCa from patients with aggressive PCa. Supplementary Table S3A: a complete list of germline mutated genes significantly associated with indolent PCa. Supplementary Table S3B: a complete list of germline mutated genes significantly associated with aggressive PCa. Supplementary Table ISN4: molecular networks enriched with germline and somatic mutations in indolent PCa. Supplementary Table ISN5: molecular networks enriched with germline and somatic mutations in aggressive PCa.
